# Linc00887 suppresses tumorigenesis of cervical cancer through regulating the miR-454-3p/FRMD6-Hippo axis

**DOI:** 10.1186/s12935-020-01730-w

**Published:** 2021-01-07

**Authors:** Pei Li, Jinsheng Wang, Lingran Zhi, Fengmei Cai

**Affiliations:** 1 Department of Obstetrics and Gynecology, Shaanxi Province Geriatric Hospital, Xi’an, 710005 China; 2 Department of Obstetrics and Gynecology, Xi’an Jingkai District Women and Children’s Hospital, Xi’an, 710000 China; 3 Pathology Department, Xi’an People’s Hospital (Xi’an Fourth Hospital), Xi’an, 710004 China

**Keywords:** Cervical cancer, linc00887, miR-454-3p, FRMD6-hippo axis, RNA-ceRNA interaction

## Abstract

**Background:**

Emerging evidence suggested that long intergenic noncoding RNA (lincRNA) 00887 (NR_024480) reduced the invasion and metastasis of non-small cell lung cancer by sponging miRNAs degradation. However, the role and regulatory mechanism of linc00887 in the progression of cervical cancer remain largely unknown.

**Methods:**

In vivo or vitro, RT-qPCR assay was used to detect the expression of linc00887 in human normal (N = 30), cervical cancer tissues (N = 30), human normal cervical epithelial cells (Ect1/E6E7) and cervical cancer cell lines (HeLa, C33A). Then, CCK-8 and Transwell assays were used to examine cell proliferation and invasion when linc00887 was overexpressed or knocked down. In addition, bioinformatics, luciferase reporter gene and pull-down assays were used to predict and validate the relationship between linc00887 and miR-454-3p. Moreover, we detected the expression of miR-454-3p in Ect1/E6E7, HeLa and C33A cells when linc00887 was overexpressed or knocked down. Cell proliferation and invasion were also measured when pcDNA-linc00887 and miR-454-3p were transfected alone or together. Next, miR-454-3p target gene was predicted and validated by bioinformatics and luciferase reporter gene assays. Gain- and loss-of-function experiments were performed in HeLa cells to evaluate the effect of miR-454-3p or linc00887 on the expression of FERM domain containing protein 6 (FRMD6) protein and several key proteins in the FRMD6-Hippo signaling pathway.

**Results:**

Linc00887 was downregulated in cervical cancer tissues or human cervical cancer cell lines (Hela, C33A) compared with normal tissues or cell lines. Overexpression of linc00887 inhibited proliferation and invasion HeLa and C33A cells, while linc00887 knockdown had the opposite effect. Linc00887 bound with miR-454-3p, and overexpression of miR-454-3p rescued linc00887-induced inhibition proliferation and invasion of HeLa cells. MiR-454-3p targeted and suppressed the expression of FRMD6, and linc00887 suppressed tumorigenesis of cervical cancer through activating the FRMD6-Hippo signaling pathway.

**Conclusions:**

Linc00887, sponging miR-454-3p, inhibited the progression of cervical cancer by activating the FRMD6-Hippo signaling pathway.

## Background

Cervical cancer is a common female malignant tumor [1], and there is no effective treatment at present. It is reported that human papillomavirus (HPV) infection is the key to cervical cancer [2], and the E6/E7 protein encoded by HPV is the key to triggering cervical cancer [3]. The estimates of cervical cancer deaths were about 30,400 in China in 2014, with a crude mortality rate of 4.57/100,000 [4]. In 2018, around 570,000 women worldwide suffered from cervical cancer, 311,000 women died of cervical cancer, the standard incidence rate was 13.1/100,000 in all ages, and the standardized mortality rate was 6.9/100,000 in all ages [5]. Cervical intraepithelial neoplasia (CIN) is a sign of cervical lesions, and screening can help the treatment of cervical cancer. According to the World Health Organization (WHO), CIN can be divided into three types: CIN1 (mild), CIN2 (moderate), and CIN3 (severe). The CIN1 infection only needs to be observed, and the CIN2 and CIN3 infections require special treatment. Previous studies reported that pretreatment of cervical cancer cells with proteasome inhibitor MG132 promotes bystander killing mediated by chemotherapy drug mitomycin C [6]. Bevacizumab, an antiangiogenic agent targeting vascular endothelial growth factor 2 (VEGF-2), is added to standard chemotherapy for cervical cancer to improve survival [7, 8]. Chemotherapy is mainly used to treat advanced or recurrent cervical cancer, but the prognostic effect of chemotherapy does not reach the expected effect. Therefore, early prevention and treatment of cervical cancer is particularly important.

In recent years, it has been reported that lncRNAs play important roles in the pathogenesis and development of cervical cancer [9, 10]. It was reported that lncRNA antisense non-coding RNA in the INK4 locus (ANRIL) promotes cervical cancer carcinogenesis via the PI3K/Akt pathway [11], and lncRNA colorectal neoplasia differentially expressed (CRNDE) enhances cervical cancer progression by suppressing expression of p53 upregulated modulator of apoptosis (PUMA) [12]. Moreover, some lncRNAs inhibit the development of cervical cancer. LncRNA Growth arrest special 5 (GAS5) inhibited the growth and metastasis of cervical cancer cells [13]. Linc00887 (NR_024480.1), located at chr3q29, has a length of 3021 nucleotides and consists of three exons. Previous studies showed that linc00887 played an anti-cancer role in lung cancer [14]. In addition, it has been reported that linc00887 plays an important role in nasopharyngeal carcinoma, non-small cell lung cancer and renal cell carcinoma [15, 16], but the role of linc00887 was largely unknown in cervical cancer. We recently performed a transcriptome sequencing combined with mRNA-miRNA-lncRNA network analysis, which revealed that linc00887 was distinctly downregulated in cervical cancer tissues and displayed a strongly positive relation with multiple molecular components in the tumor suppressor pathway Hippo/TAZ, including MST1, MST2, and TAZ etc. (the results will be published in another paper). Therefore, we aim to explore the role and mechanism of linc00887 in cervical cancer and provide a new perspective for the treatment of cervical cancer.

MicroRNAs (miRNAs) are endogenous non-coding RNAs that bind to 3′-untranslated regions (3′-UTRs) of mRNAs, resulting in the inhibition of protein translation or mRNA degradation. MiR-454-3p plays a dual role in cancer progression. It was reported that miR-454-3p level was downregulated in glioma [17], while miR-454-3p promoted the development of non-small cell carcinoma [18]. However, one report revealed that miR-454-3p enhanced cell proliferation and induced apoptosis in human cervical cancer by targeting TRIM3 [19]. All these studies demonstrated that miR-454-3p acts important roles in the tumorigenesis and progression of various cancers. Thus, a better understanding of the disorder of miR-454-3p may help to better understand the progression of cervical cancer and improve the treatment of cervical cancer.

Here, we demonstrated that linc00887 was remarkably down-regulated in human cervical cancer tissues or cell lines. Overexpression of linc00887 inhibited the proliferation and invasion of HeLa and C33A cells, while linc00887 knockdown had the opposite effect. Bioinformatics, luciferase reporter gene and pull-down assays were predicted and verified that linc00887 bound to miR-454-3p. Moreover, we found that linc00887 overexpression inhibited miR-454-3p level in Hela cells. In addition, we investigated that linc00887 enhanced cell proliferation and invasion by regulating miR-454-3p expression. FERM domain containing protein 6 (FRMD6) was confirmed as the target gene of miR-454-3p. In all, our research revealed that linc00887 inhibited cervical cancer cell proliferation and invasion by adsorbing miR-454-3p and upregulating FRMD6 expression.

## Materials and methods

### Patient tissue samples

In this study, 30 female patients (49 ± 6.1 years old) were diagnosed with cervical cancer in Shaanxi Province Geriatric Hospital (Xi’an, China) without medical treatment or surgical treatment. Cervical cancer tissues and corresponding normal tissues were collected in Shaanxi Province Geriatric Hospital during April 2017 and May 2018. All patients received informed consent. This study was approved by the Ethical Review Board for Research in the Shaanxi Province Geriatric Hospital. The tissue samples were stored in liquid nitrogen at − 80 °C.

### Cell lines and cell culture

Immortalized human cervix squamous cells (Ect1/E6E7 Cat.#AC39960) and cervical cancer cell lines (HeLa Cat.#TCHu187, C33A Cat.#BNCC337882, Caski Cat.#BNCC338223, ME180 Cat.#CC1107, Siha Cat.#TCHu113) were purchased from American Type Culture Collection (ATCC, Manassas, VA) or Cell Bank of Type Culture Collection of the Chinese Academy of Sciences, Shanghai Institute of Cell Biology (Shanghai, China). Ect1/E6E7 cells were cultured in MEM (minimal essential medium), and cervical cancer cell lines were cultured in DMEM (Dulbecco’s modified Eagle's medium). These media all supplemented with 10% fetal bovine serum (FBS, Gibco, Grand Island, NY, USA), and cells were all cultured in a humidified incubator with 5% CO_2_ at 37 °C.

### Cell transfection

Linc00887 siRNA, FRMD6 siRNA and scrambled siRNA (scramble), miR-454-3p mimic, NC mimic were synthesized and purchased from Geneseed Biotech (Guangzhou, China). The full-length of linc00887 sequence was purified and ligated into pcDNA™ 3.1 vector (Thermo Fisher Scientific, Waltham, MA, USA), referred as pcDNA-linc00887. The empty pcDNA3.1 vector (Vector) was used as a negative control. When the cell density reached approximately 70% confluence, the cells were seeded in a six-well plate of 1.2 × 10^5^ cells/well. Transfection of linc00887 siRNA, FRMD6 siRNA or 1 μg/mL pcDNA-linc00887 was carried out with Lipofectamine™ 3000 (Invitrogen, Carlsbad, CA, USA) according to the manufacturer's instructions.

### Reverse transcription-qPCR (RT-qPCR)

According to the manufacturer's instructions, Trizol reagent (Invitrogen, Carlsbad, CA, USA) was used to extract the total RNA of cells and tissues, and the purity and integrity of the total RNA were tested. We reverse transcribed linc00887 into cDNA using the first strand synthesis system of superscript III (Thermo Fisher, Waltham, Ma, USA). Next, quantitative PCR was used to detect the specificity and amplification efficiency of primers. In the formal test, the total volume was 15 μL, including 6.5 μL sterile water, 7.5 μL SYBR Green PCR master mix, 0.5 μL upstream primer and 0.5 μL downstream primer. According to the manufacturer's instructions, U6 or GAPDH was taken as the control. Gene expression was identified by commercial kit SYBR ® premix dimer eraser Kit (Takara, Dalian, China). The PCR procedure was as follows: 95 °C 1 min, 1 cycle; 95 °C 20 s, 56 °C 1 s, 72 °C 15 s, 35 cycles. The relative quantification was determined by the 2^−ΔΔCt^ method. The primer sequences are as follows: linc00887 forward: 5′- ATC CAA GGA CTT GTG CTG GG-3′ and reverse: 5′-TGC TGA GCT GCT TCT TGG AA-3′; U6 forward: 5′-TGC GGG TGC TCG CTT CGG CAG C-3′ and reverse: 5′-CCA GTG CAG GGT CCG AGG T-3′; GAPDH 5′-TTG GTA TCG TGG AAG GAC TCA-3′ and reverse: 5′-TGT CAT CAT ATT GGC AGG TT-3′; FRMD6 forward: 5′-TGA CAC GCC ATA CAC AAG CT-3′ and reverse: 5′-CTT TGG CCT CAG ACT GAG CA-3′.

### Cell viability assay

The 96 well plates were equipped with 100 μL cell suspension/well, and then the culture plate was cultured in the incubator for 24 h in 37 °C with 5% CO_2_. 10 μL of substance to be tested was added to the culture plate, and the culture plate was incubated in the incubator for 24 h, 48 h and 72 h, respectively. 10 μL of CCK-8 solution (Dojindo Laboratories, Japan) was added to each hole, and then the culture plate was incubated in the incubator for 1 h. Finally, the absorbance at 450 nm was measured with a microplate reader.

### Transwell invasion assay

The 8.0-μm chamber plate was used to detect cell invasiveness, and the transwell filter was required to be equipped with Matrigel (BD, New Jersey, USA). At the beginning, the cell suspension was added to the transwell chamber, and then 300 μL of serum-free DMEM medium was added to the upper chamber plate, and 500 μL of DMEM medium containing 10% FBS was added to the lower chamber plate. After cells were cultured for 48 h, the chamber was carefully removed with tweezers. When the upper chamber liquid was dried, it was transferred to the air and added 500 μL of methanol, and then it fixed at 25 °C for 30 min. Take out the chamber, dry the upper chamber fixing solution, transfer it to the pore pre added with about 500 μL of crystal violet solution, and dye at room temperature for 30 min. After washing with clear water, take out the chamber and suck the upper chamber liquid. Then, wipe the cells on the surface of the membrane at the bottom of the upper chamber carefully with a wet cotton stick and take out the membrane with forceps, and then dry it and move it onto the slide, and seal it with neutral glue. Finally, 10 fields of vision were randomly selected under Olympus IX70 inverted microscope (Olympus Corp., Tokyo, Japan), and the experimental results were counted.

### Dual fluorescein reporter gene assay

StarBase (http://StarBase.sysu.edu.cn/index.php) was used to predict potential binding sites between linc00887 and miR-454-3p. To further identify potential relationships, we performed a luciferase report analysis. We cloned miR-454-3p and linc00887 wild-type and mutant binding sites into pGL3 promoter vectors to construct luciferase reporter vector. HEK-293 T cells were seeded into 24-well plates, and when it reached to 70% confluence, wild-type or mutant reporter vectors and miR-454-3p mimic or NC-mimic were co-transfected into cells by using Lipofectamine 3000 (Invitrogen, Carlsbad, CA, USA). After transfection for 48 h, cells were harvested and analyzed using a dual luciferase reporter kit (Promega, Madison, Wisconsin, USA) to normalize luciferase reporter activity to Renilla luciferase activity.

### RNA pull-down assays

In order to confirm the relationship between linc00887 and miR-454-3p, biotin-labeled miR-454-3p was synthesized and transfected into HEK293T cells. Bio-miR-454-3p probe was transcribed and purified by GenePharma Company (Shanghai, China) through the AmpliScribe™ T7-Flash™ Biotin-RNA Transcription Kit (Epicenter, Madison, Wisconsin, USA). After 48 h, the cells were washed and lysed, and then the extract was incubated with anti-rabies magnetism at 4 °C for 3 h. Then, the beads were washed twice with ice-cold buffer, three times with low salt buffer, and once with high salt buffer. Finally, the RNA-RNA complex was eluted and the RNA pull-down product was detected by specific RT-qPCR.

### Western blotting

Firstly, the total protein of cells was extracted. The cell medium was poured out and absorbed on absorbent paper. We added 3 mL of 4 °C precooled PBS (0.01 M pH 7.2 ~ 7.3) to each bottle of cells and washed the cells three times, and then added 400 μL of lysate containing PMSF (100 mM) to each bottle of cells and lysed on ice for 30 min. After lysis, the cell fragments and lysate were transferred to a 1.5 mL centrifuge tube and centrifuged at 12,000 rpm at 4 °C for 5 min. Then, the supernatant was quantified with BCA protein detection kit (Shanghai Institute of Biotechnology). The protein sample was then loaded onto 10% sulfate polyacrylamide gel electrophoresis (SDS-PAGE) (electrophoresis time 4–5 h, voltage 40 V), until bromo phenol blue ran out to stop electrophoresis and transferred to nitrocellulose membrane (Millipore, Boston, MA, USA). After sealing with 5% skim milk, the imprinting was detected with a specific antibody. After washing with PBS, Goat anti-rabbit IgG H&L (ab205718; 1:1000) was added. The main antibodies used in this study include Rabbit anti-FRMD6/willin antibody (ab110675) (1:500), Rabbit anti Mst1/MST2 antibody (ab87322; 1:300), Rabbit anti LATS1 + LATS2 (phospho T1079 + T1041) antibody (ab111344; 1:300), Rabbit anti-TAZ antibody (ab84927; 1:400), Rabbit anti-TIMP-1 (ab211926; 1:400), Rabbit anti-TIMP-2 (ab180630; 1:400), Rabbit anti-MMP-2 (ab92536) and anti-MMP-9 (ab76003). After diluting the first antibody to the appropriate concentration with TBST, the antibody was added to the fresh-keeping film, incubated at 25 °C for 1–2 h and eluted three times. In the same way, the second antibody diluent was prepared to contact with the membrane, incubated at room temperature for 1–2 h, eluted three times, and then chemiluminescence reaction was carried out. Next, A and B reagents were directly mixed in equal volume, and then mixed with the membrane to remove the residual liquid and put into the X-ray clip. In the darkroom, pour 1 × developer and fixer into the plastic disk respectively, and conduct exposure development under the red light. Finally, the film was scanned or photographed, and the molecular weight and net optical density of the target were analyzed by the GEL Image processing system.

### Statistical analysis

The data were represented by mean ± standard error of mean (SEM) and processed by SPSS 22.0 software (IBM Corp., Armonk, NY, USA). The student t-test was used to compare the significance between two groups, while the analysis of variance was used to test the differences of three groups or more groups. *P* < 0.05 was statistically significant. All experiments were repeated at least three times.

## Results

### The expression of linc00887 was downregulated in cervical cancer tissues or cell lines

**Fig. 1:**
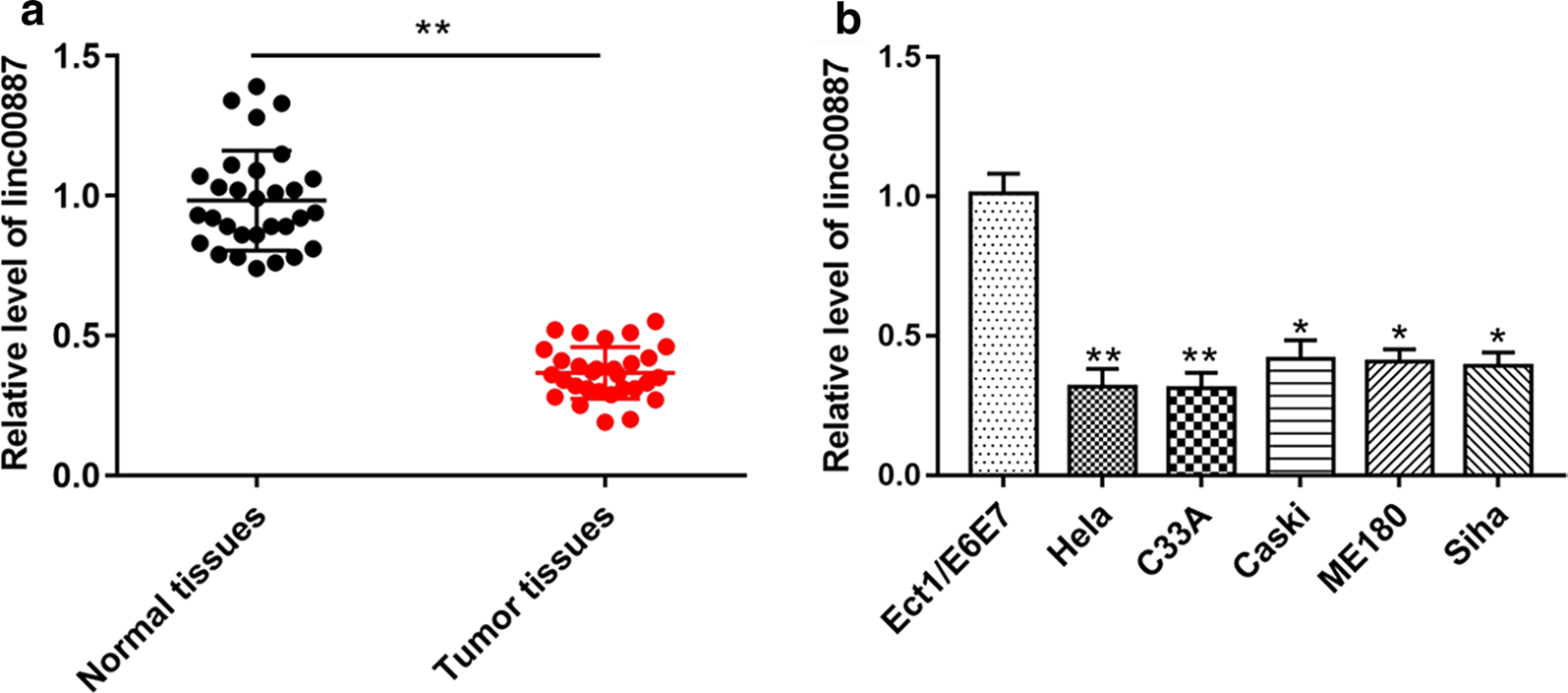
The expression of linc00887 was presented in cervical cancer tissues and cells line. **a** The relative expression of linc00887 in cervical cancer tissues of cervical cancer patients (n = 30) and normal tissues of donors (n = 30) was detected by RT-qPCR. **p < 0.01 versus tumor tissues. Paired t-test was used to analyze the differences of data. **b** Linc00887 level in cervical cancer cell lines (Hela, C33A, Caski, ME180 and Siha) was detected by RT-qPCR, compared with normal cancer cell lines (Ect1/E6E7). *p < 0.05 versus Ect1/E6E7 cell lines, **p < 0.01 versus Ect1/E6E7 cell lines

In order to investigate the role of linc00887 in cervical cancer, we detected the linc00887 level in human cervical cancer tissues and cell lines. As shown in Fig. 1a, the result of RT-qPCR showed that the expression of linc00887 was downregulated in tumor tissues compared with normal tissues (*p* < 0.01). Next, we selected five cervical cancer cell lines (Hela, C33A, Caski, ME180 and Siha) to examine the linc00887 level in vitro, which were detected by RT-qPCR. The results showed that linc00887 level was also downregulated in cervical cancer cell lines, especially in HeLa and C33A cell lines, compared with normal cervical cell lines (Ect1/E6E7) (*p* < 0.05) (Fig. 1b). Our results suggested that linc00887 might be a new biomarker in cervical cancer progression.

### Overexpression of linc00887 led to a significant inhibition of cell proliferation and invasion, and knockdown of linc00887 led to a significant promotion of cell proliferation and invasion

**Fig. 2:**
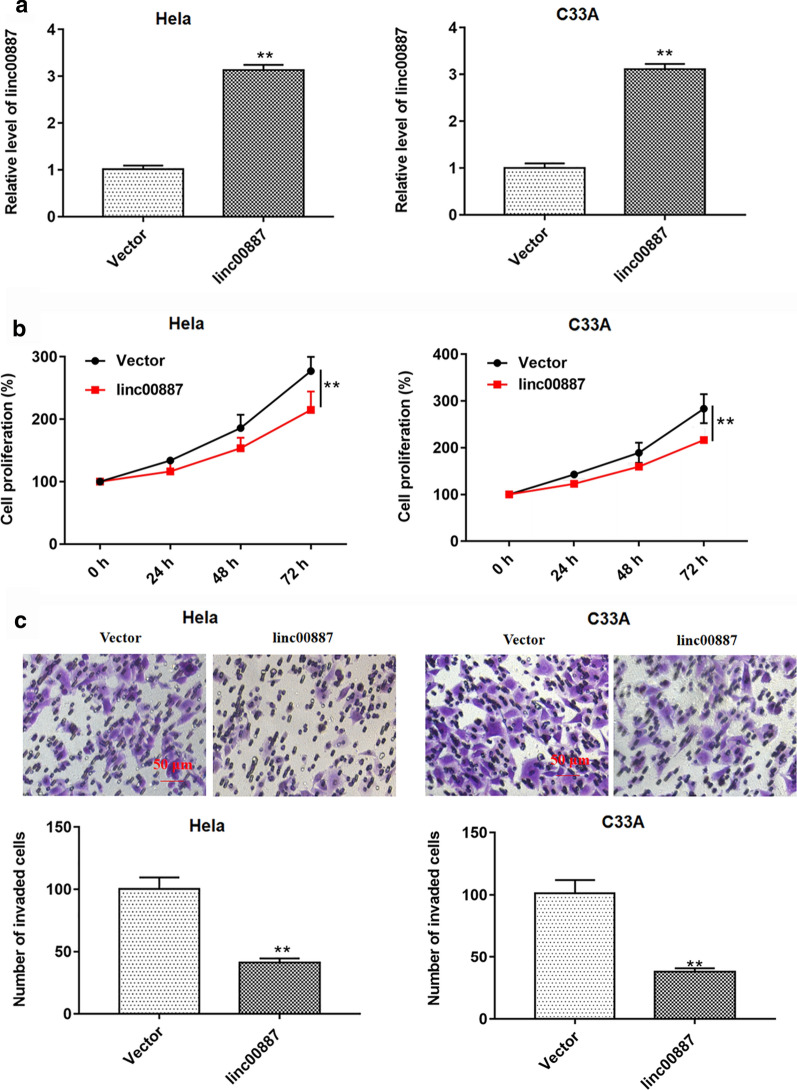
Effect of linc00887 overexpression on cell proliferation and invasion. HeLa or C33A cells were transfected with pcDNA-linc00887 (linc00887) or pcDNA3.1 (Vector). **a** RT-qPCR was used to detect linc00887 level in Hela or C33A cell supernatant. **b** CCK-8 assay was used to detect cell proliferation after culturing for 0, 24, 48 and 72 h. **c** Cell invasion was performed by Transwell assay. **p < 0.05 versus Vector

**Fig. 3:**
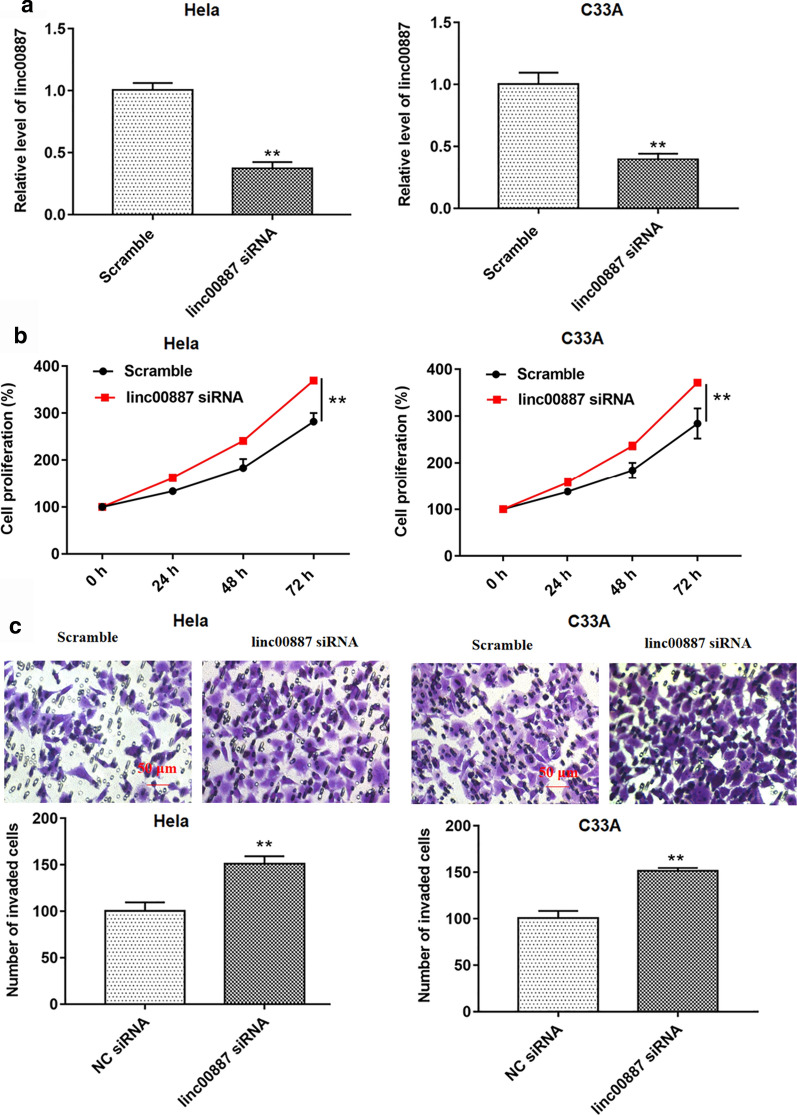
Effect of linc00887 knockdown on proliferation and invasion in HeLa or C33A cell line. HeLa or C33A cells were transfected with linc00887 siRNA or scramble. **a** Linc00887 level in Hela or C33A cell supernatant was examined by RT-qPCR. Cell proliferation (**b**) was detected by CCK-8 assay and cell invasion (**c**) was examined by Transwell assay. **p < 0.05 versus scramble

Then, we tested the effect of linc00887 on cell behavior in vitro. Firstly, we transfected the pcDNA3.1 empty vector (Vector), pcDNA-linc00887, scramble and linc00887 siRNA into HeLa cells, respectively. Then, linc00887 level was detected by RT-qPCR. Compared with vector group, transfection with pcDNA-linc00887 enhanced linc00887 level in HeLa or C33A cells, while transfection with linc00887 siRNA inhibited the expression of linc00887 in HeLa and C33A cells (Figs. 2a and 3a). The results of CCK-8 assay and Transwell assay revealed that linc00887 overexpression inhibited cell proliferation and invasion (Fig. 2b, c), while linc00887 knockdown enhanced cell proliferation and invasion (Fig. 3b, c). TIMP-1, TIMP-2, MMP-2 and MMP-9 protein levels could reflect the invasiveness of cells. The increase of TIMP-1 and TIMP-2 protein levels led to the decrease of cell invasion, and the increase of MMP-2 and MMP-9 protein levels had the opposite effect, which is consistent with our previous results that overexpression of linc00887 inhibited cell invasion, while knockdown of linc00887 enhanced cell invasiveness (Additional file 1: Figure S1). These results proved that linc00887 could inhibit cell proliferation and invasion in vitro.

### Linc00887 bound to miR-454-3p in cervical cells, and miR-454-3p overexpression rescued the inhibition of linc00887 on cell proliferation and invasion

**Fig. 4:**
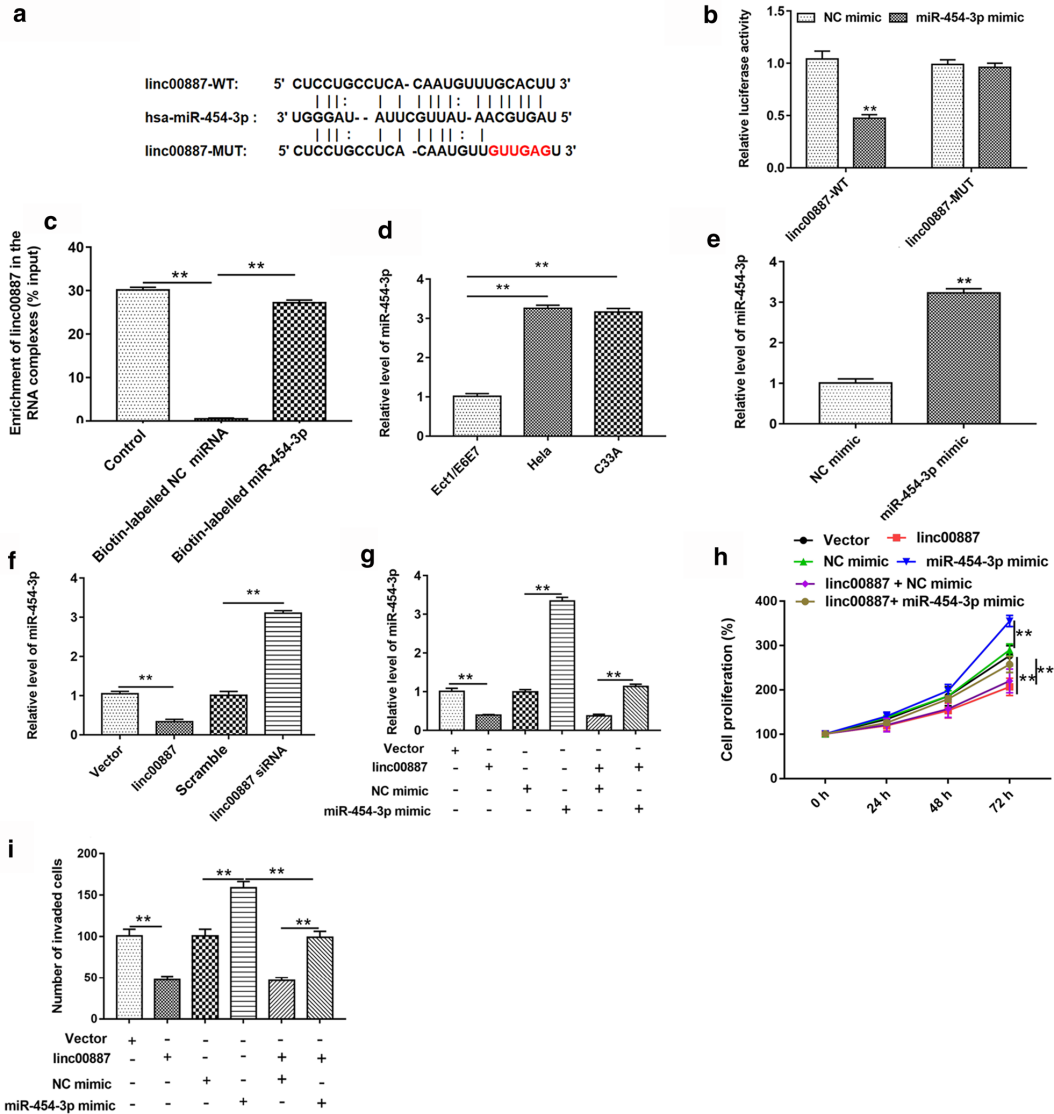
Linc00887 bound to miR-454-3p, and miR-454-3p overexpression rescued the inhibition of pcDNA-linc00887 on cell proliferation and invasion. **a** Starbase online (http://starbase.sysu.edu.cn/) was used to predict the relationship of wild-type linc00887 or mutant linc00887 with has-miR-454-3p. Dual fluorescein report gene assay (**b**) and Pull down assay (**c**) were used to verify the relationship between linc00887 and miR-454-3p. **p < 0.01 versus NC mimic or Biotin-labelled NC miRNA. **d** MiR-454-3p level was detected in immortalized squamous cells of human cervix (Ect1/E6E7) and cervical cancer (Hela and C33A) cell lines. **p < 0.01 versus Ect1/E6E7. **e** RT-qPCR was used to detect that the effect of miR-454-3p overexpression on miR-454-3p expression. **P < 0.01 versus NC mimic. **f** Effect of overexpression of linc00887 on miR-454-3p expression and knockdown of linc00887 on miR-454-3p expression. **p < 0.01 versus Vector or scramble. **g** Effect of pcDNA-linc00887 vector was transfected into Hela cells together with miR-454-3p mimic vector on miR-454-3p expression. **p < 0.01 versus Vector, NC mimic or pcDNA-linc00887 together with miR-454-3p mimic. **h**, **i** Effect of pcDNA-linc00887 vector was transfected into Hela cells together with miR-454-3p mimic vector on cell proliferation and invasion. **p < 0.01 versus Vector, NC mimic or pcDNA-linc00887 together with miR-454-3p mimic

To forecast the downstream target miRNA of linc00887, Starbase online (http://starbase.sysu.edu.cn/) was used to search potential target miRNAs of linc00887. The output showed that there was a binding site between miR-454-3p and wild type of linc00887 (Fig. 4a). Then, dual fluorescein reporter gene and pull-down assay were used to verify the relationship between linc00887 and miR-454-3p (Fig. 4c). These results revealed that linc00887 bound to miR-454-3p. In order to investigate the role of miR-454-3p in cervical cancer, we examined the miR-454-3p level in HeLa and C33A cell, and we found that miR-454-3p level was upregulated in HeLa and C33A cell lines, compared with immortalized human cervix squamous cells (Ect1/E6E7) (Fig. 4d). In addition, we used the miR-454-3p mimic to overexpress the miR-454-3p level (Fig. 4e). Next, we explored the effect of linc00887 on miR-454-3p level in cervical cancer. These results showed that overexpression of linc00887 inhibited the miR-454-3p level, and linc00887 knockdown enhanced the miR-454-3p level (Fig. 4f). And miR-454-3p could reversed the inhibition of miR-454-3p level induced by linc00887 (Fig. 4g). Moreover, we detected the cell proliferation and invasion, and we found miR-454-3p could reversed the inhibition of cell proliferation and invasion induced by linc00887 (Fig. 4h, i). These results suggested that linc00887 might act by sponging and inhibited the expression of miR-454-3p.

### MiR-454-3p inhibitor reversed linc00887 knockdown induced expression of miR-454-3p and cell proliferation/invasion

**Fig. 5:**
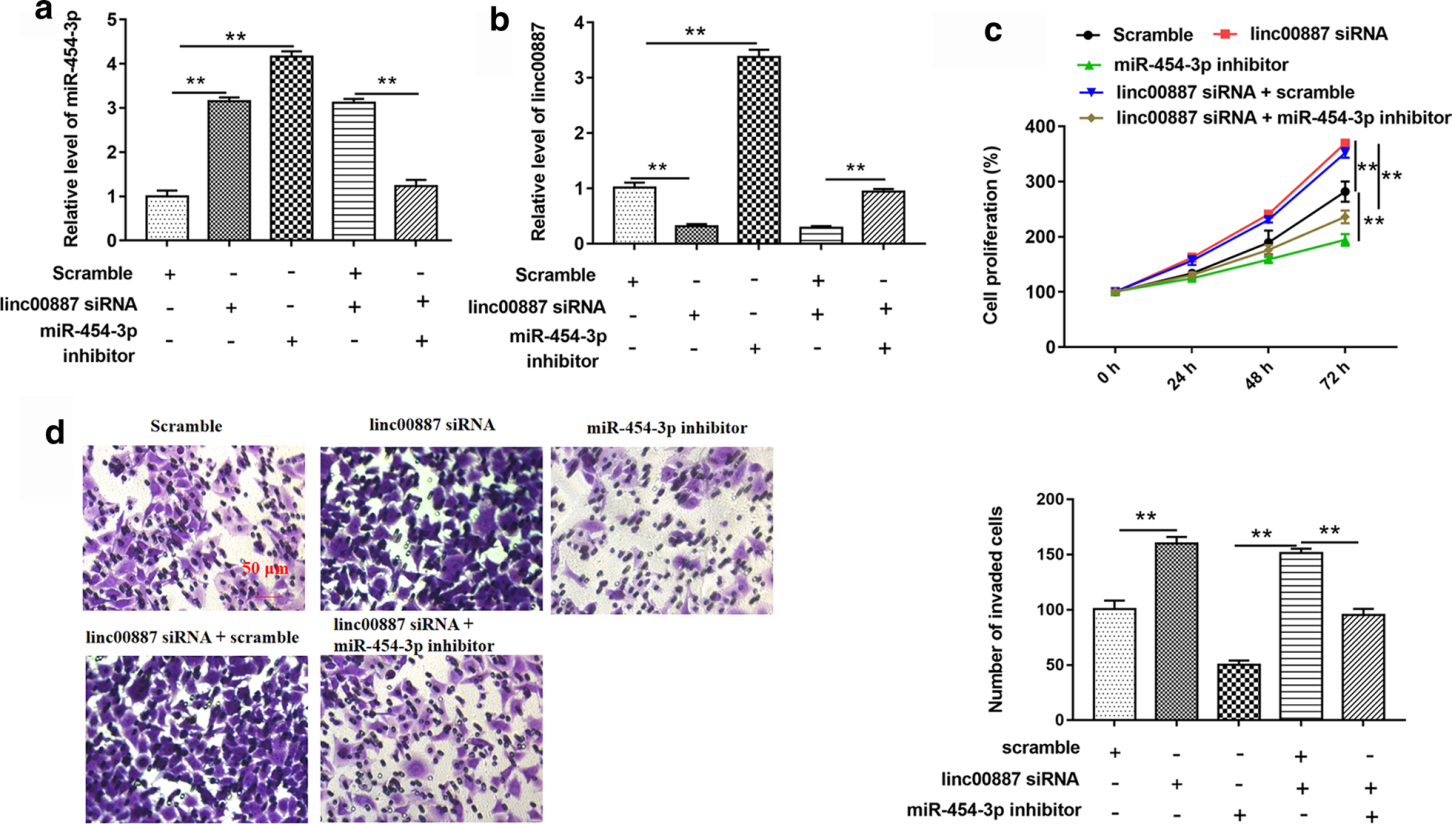
Effect of miR-454-3p inhibitor vector was transfected into Hela cells together with linc00887 knockdown on expression of miR-454-3p, linc00887, cell proliferation and invasion. Scramble alone, linc00887 siRNA vector alone, miR-454-3p inhibitor, linc00887 siRNA vector together with scramble and linc00887 siRNA vector together with miR-454-3p inhibitor were transfected into HeLa cells. **a** Effect of these five vectors transfected into Hela cells on miR-454-3p expression, which was detected by RT-qPCR. **b** RT-qPCR was used to detect that effect of these five vectors transfected into Hela cells on linc00887 expression. **c**, **d** Effect of these four vectors transfected into Hela cells on cell proliferation and invasion, which was detected by CCK-8 assay and Transwell assay. **p < 0.01 versus Scramble alone or linc00887 siRNA together with scramble

In order to investigate the effect of linc00887 knockdown on miR-454-3p level, linc00887 siRNA and miR-454-3p inhibitor were transfected alone or together into HeLa cells. As shown in Fig. 5a, the result of RT-qPCR revealed that miR-454-3p level was upregulated in HeLa cells after linc00887 siRNA transfected into cells alone, compared with scramble group. On the contrary, miR-454-3p inhibitor reversed the increase of miR-454-3p level induced by linc00887 knockdown, and miR-454-3p inhibitor also antagonized the decrease of linc00887 level induced by linc00887 siRNA (Fig. 5a, b). In addition, we found that miR-454-3p inhibitor reversed the facilitation of cell proliferation and invasion induced by transfection with linc00887 siRNA (Fig. 5c, d). These data combined with Fig. 4 indicated that linc00887 and miR-454-3p negatively regulate each other.

### FRMD6 bound to miR-454-3p, and FRMD6 level was downregulated in cervical cancer cells

**Fig. 6:**
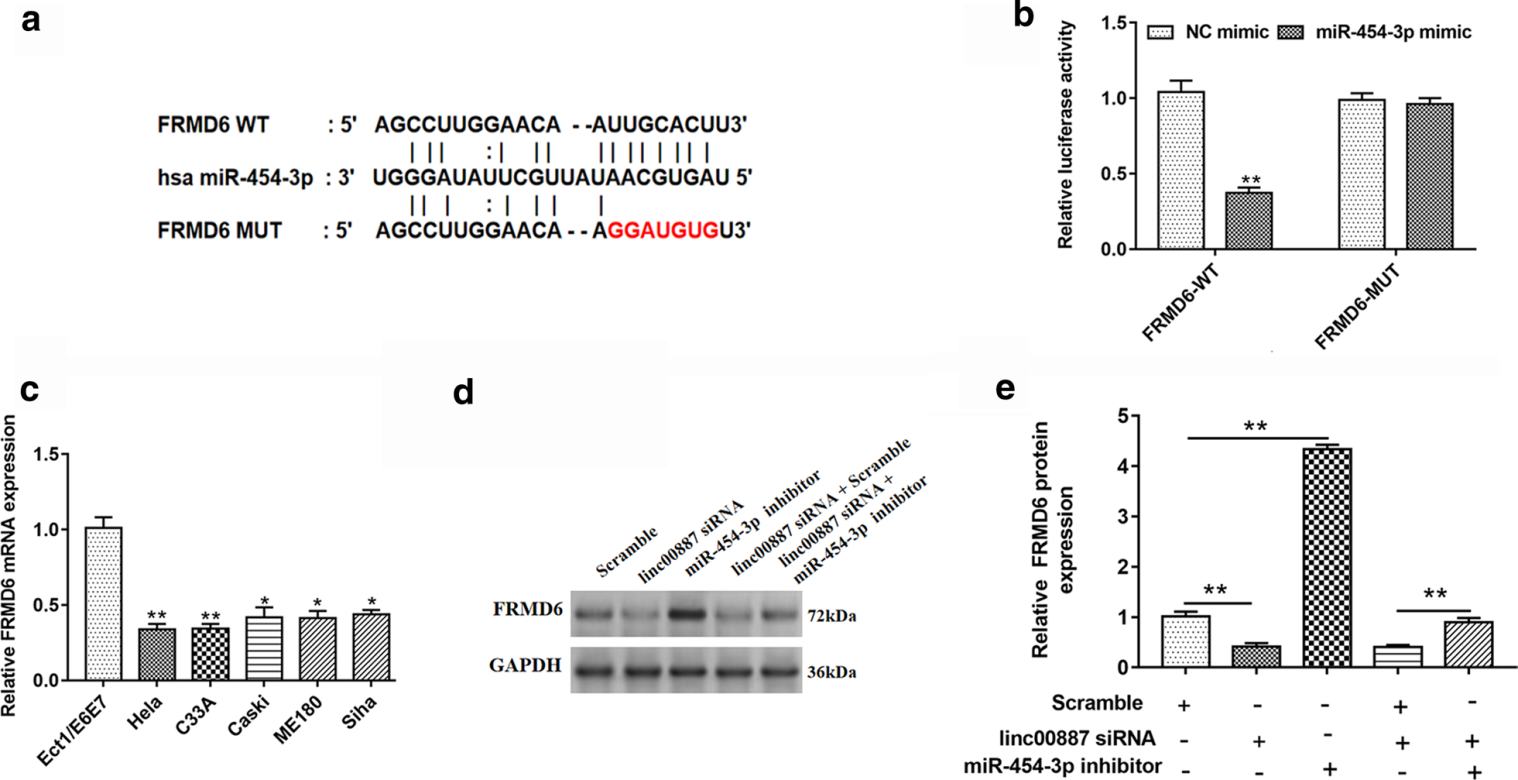
FRMD6 bound to miR-454-3p, and the expression of FRMD6 mRNA was detected in cervical cancer cell lines. **a** Starbase online (http://starbase.sysu.edu.cn/) was used to predict the relationship of wild type FRMD6 or mutant FRMD6 with has-miR-454-3p. **b** Dual fluorescein report gene assay was used to verify the relationship of wild type FRMD6 or mutant FRMD6 with has-miR-454-3p in HEK293 cells. **p < 0.01 versus NC mimic. **c** Relative FRMD6 mRNA expression was detected in Ect1/E6E7, Hela, C33A, Caski, ME180 and Siha cell lines. **p < 0.01 versus Ect1/E6E7 cell line. **d**, **e** Effect of linc00887 siRNA transfected into Hela cell line together with miR-454-3p inhibitor on FRMD6 protein expression, which was detected by Western blot assay. Histogram (**e**) was the quantization of graph D. **p < 0.01 versus scramble alone or linc00887 siRNA together with scramble

To search the molecular target of miR-454-3p, Starbase (http://starbase.sysu.edu.cn/) was used to predict potential genes. We found that the gene of FRMD6 might be regulated by miR-454-3p, and the binding site of FRMD6 mRNA 3′-UTR and miR-155-5p was shown in Fig. 6a. In addition, the result of luciferase reporter gene assay confirmed that FRMD6 could bind to miR-454-3p (Fig. 6b). Next, we examined the FRMD6 mRNA level in cervical cancer cells, and we found that FRMD6 mRNA was downregulated in HeLa, C33A, Caski, ME180, Siha cells, compared with Ect1/E6E7 cells (Fig. 6c). Moreover, we explored the regulation of linc00887 and miR-454-3p on FRMD6 protein level. These results confirmed that linc00887 knockdown inhibited the FRMD6 protein level, which could be rescued by miR-454-3p inhibitor (Fig. 6d, e). These results suggested linc00887 upregulated the FRMD6 protein level by negatively regulating miR-454-3p.

### Overexpression of FRMD6 enhanced expression of key proteins in FRMD6-Hippo signaling pathway, and it inhibited cell proliferation and invasion

**Fig. 7:**
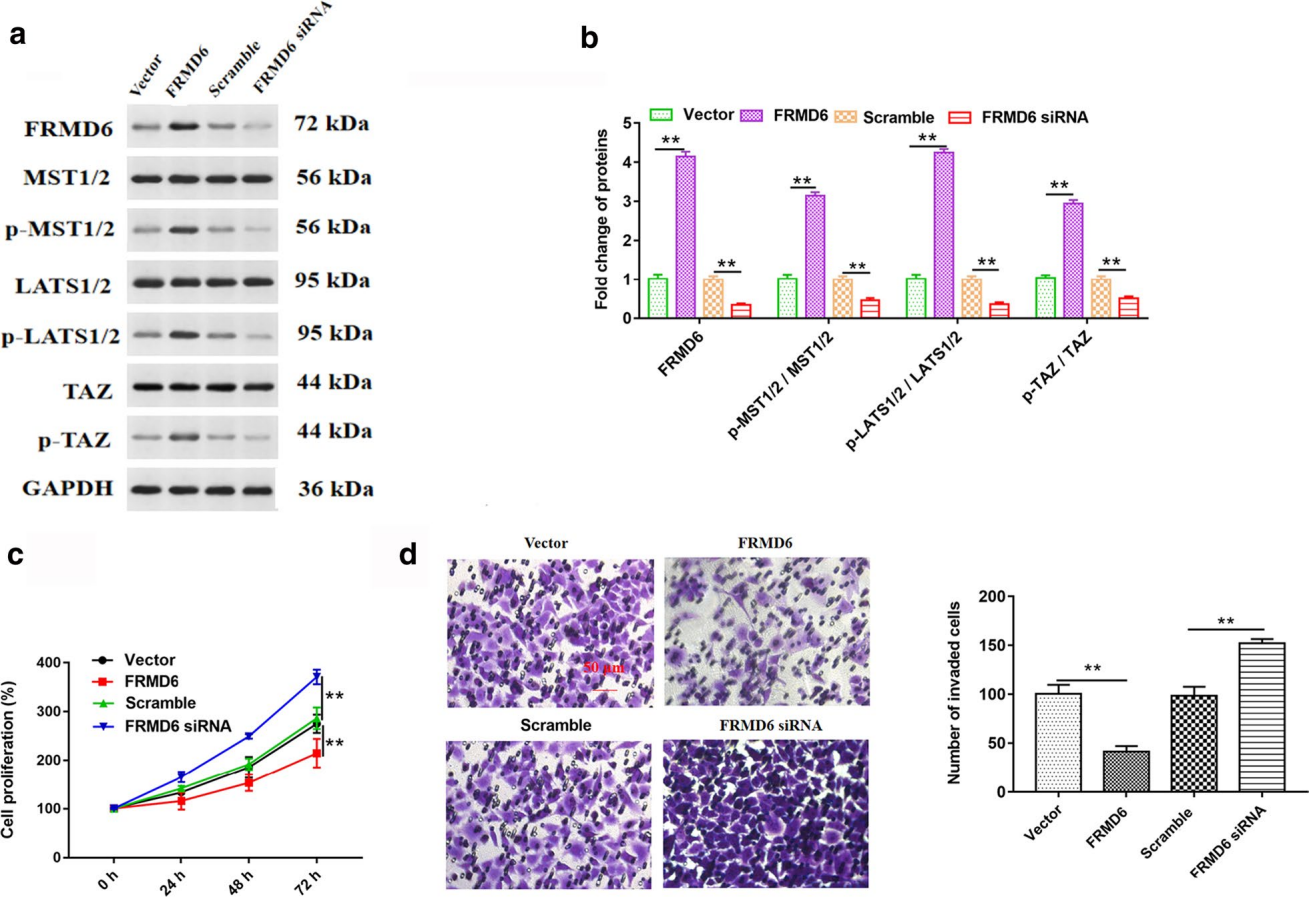
Effect of FRMD6 overexpression on the FRMD6 protein, phosphorylation of MST1/2, LATS1/2 and TAZ proteins and cell proliferation and invasion. The pcDNA3.1 empty vector, pcDNA-FRMD6 expression vector, scrambled siRNA and FRMD6 siRNA vectors were transfected into Hela cells. **a** Western blot assay revealed the effect of FRMD6 overexpression on MST1/2 protein, LATS1/2 protein, TAZ protein, phosphorylation of MST1/2, LATS1/2 and TAZ proteins. Histogram **b** was the quantization of graph A. **p < 0.01 versus Vector or Scramble. **c**, **d** Effect of these four vectors transfected into Hela cells on cell proliferation and invasion. **p < 0.01 versus Vector or Scramble

To investigate the effect of overexpression or knockdown of FRMD6 on related proteins level in FRMD6-Hippo signaling pathway, we firstly transfected four vectors into HeLa cells including Vector (pcDNA3.1), FRMD6 (pcDNA-FRMD6), scramble and FRMD6 siRNA vectors, respectively. The result of Western blot assay revealed that FRMD6 overexpression did not change total MST1/2 protein, total LATS1/2 protein, total TAZ protein level but it increased FRMD6 protein level, the phosphorylation of MST1/2, LATS1/2 and TAZ proteins level (Fig. 7a, b). Next, we detected the cell proliferation and invasion, and we found that overexpression of FRMD6 inhibited cell proliferation and invasion, while FRMD6 knockdown enhanced cell proliferation and invasion (Fig. 7c, d). These results revealed that FRMD6 may be an important target to inhibit the invasion and proliferation of cervical cancer cells.

### MiR-454-3p overexpression reversed the promotion of linc00887 overexpression on expression of FRMD6 and phosphorylation of MST1/2, LATS1/2 and TAZ proteins

**Fig. 8:**
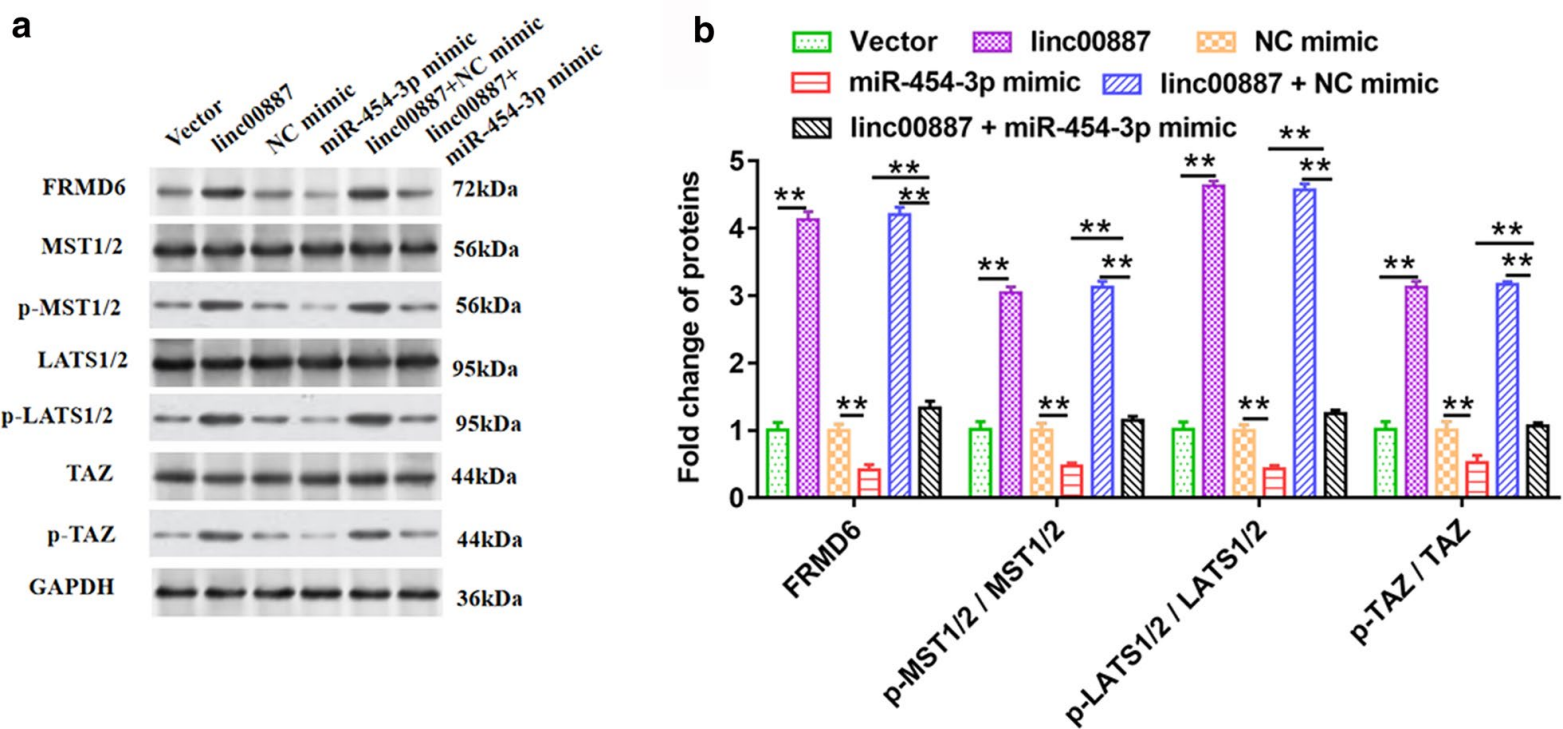
Effect of pcDNA-linc00887 transfected into Hela cells alone or together with miR-454-3p mimic on expression of FRMD6, MST1/2, LATS1/2, TAZ protein and phosphorylation of MST1/2, LATS1/2 and TAZ proteins. The pcDNA3.1 (Vector), pcDNA-linc00887, NC mimic, miR-454-3p mimic, pcDNA-linc00887 together with NC mimic, pcDNA-linc00887 together with miR-454-3p mimic were transfected into HeLa cells, respectively. **a** Effect of these six vectors on expression of FRMD6, MST1/2, LATS1/2, TAZ protein and phosphorylation of MST1/2, LATS1/2 and TAZ proteins. Histogram (**b**) was the quantization of graph A. **p < 0.01 versus Vector alone, NC mimic, miR-454-3p mimic or linc00887 together with NC mimic

**Fig. 9:**
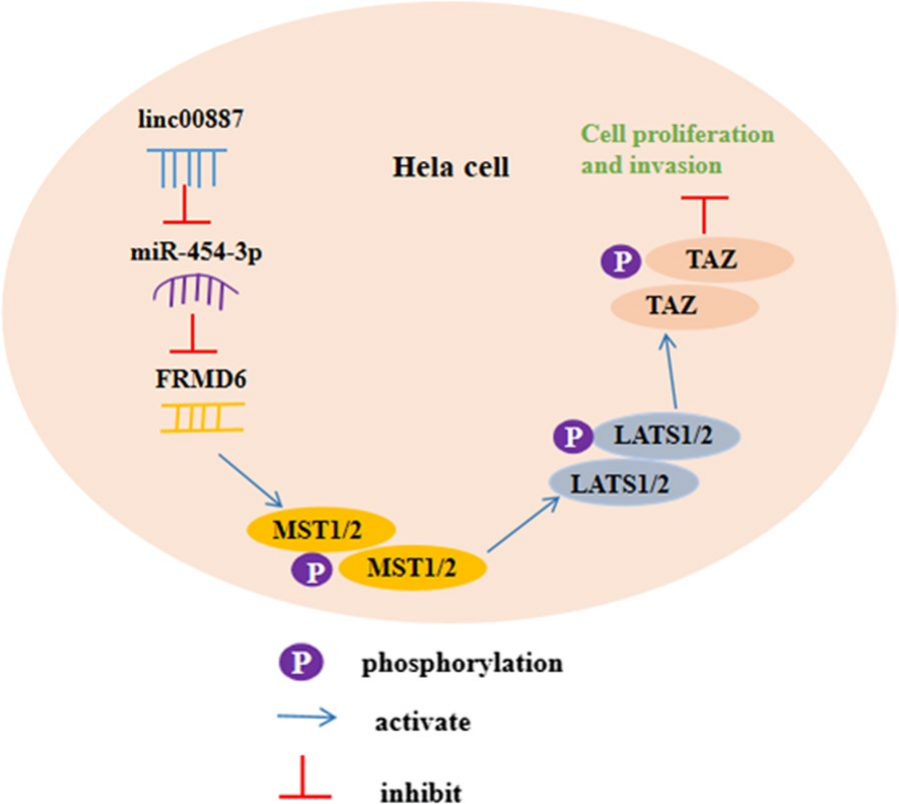
A schematic diagram for the functions and mechanism of linc00887 in regulation of cervical cancer. Linc00887 is downregulated in human cervical cancer cells in vitro, which sponges miR-454-3p and positively regulates expression of FRMD6 gene. FRMD6 is an identified regulator to phosphorylate MST1/2 and activate its downstream key kinases in Hippo signaling pathway LATS1/2 or TAZ, thus inhibiting cell proliferation and invasion

In order to further understand the mechanism of linc00887 regulating the progression of cervical cancer, we detected effect of linc00887 on the expression of key proteins in FRMD6-Hippo signaling pathway. As shown in Fig. 8a, b, the results of Western blot assay detected that overexpression of linc00887 enhanced FRMD6 protein and phosphorylation of MST1/2, LATS1/2 and TAZ proteins level. In addition, we found that miR-454-3p reversed the promotion on FRMD6 protein and phosphorylation of MST1/2, LATS1/2 and TAZ proteins level induced by linc00887 overexpression. We drew a schematic diagram indicating the mechanism through which linc00887 regulates proliferation and invasion of cervical cells: linc00887 sponging miR-454-3p to upregulate expression of FRMD6 gene which can activate the MST1/2, LATS1/2 and TAZ (key components in the Hippo signaling pathway), and thus inhibiting cell proliferation and invasion in cervical cells (Fig. 9).

## Discussion

Cervical cancer is the fourth most common cancer among women in the world, with about 528,000 new cases every year. Especially in the less developed countries, this situation is more serious [20]. In addition, the prognosis of chemotherapy for cervical cancer is not significant. Therefore, investigations of the underlying mechanism of cervical cancer metastasis, including cell proliferation and invasion are urgent and could be helpful to improve the clinical outcomes of patients. Recent studies have shown that miRNAs or lncRNAs play an important regulatory role in a variety of physiological and pathological processes [21, 22], including malignant tumors. In addition, miRNAs and lncRNAs are involved in the occurrence and metastasis of several cancers. For example, lncRNA STXBP5-AS1 inhibited cervical cancer progression by targeting miR-96-5p [23], lncRNA FOXF1 Adjacent Non-Coding Developmental Regulatory RNA (FENDRR) inhibited non-small cell lung cancer progression by targeting miR-76 [24], and lncRNA HAND2 antisense RNA 1 (HAND2-AS1) inhibited colorectal cancer progression by targeting miR-1275 [25]. It was reported that linc00887 inhibited cell invasion in non-small cell lung cancer by causing the degradation of miR-1–2, miR-206 or miR-613 [14]. In our research, we revealed that linc00887 level was downregulated in tumor tissues of patients with cervical cancer or cervical cancer cell lines, compared with epithelial cell line normal tissues or normal epithelial cell line.

Recent studies have shown that lncRNAs help to inhibit the growth and metastasis of many tumors. Linc00887 was confirmed that it was important to inhibit non-small cell lung cancer progression by regulating fibronectin 1, MET proto-oncogene, receptor tyrosine kinase and mothers against decapentaplegic homolog 4. In order to investigate the roles of linc00887 in the progression of cervical cancer cells, Hela or C33A cells were transfected with pcDNA-linc00887 to increase or decrease linc00887 level. These results revealed that linc00887 was downregulated in cervical cancer cells, and linc00887 inhibited cell proliferation and invasion, while knockdown of linc00887 had the opposite effect. Our results confirmed that linc00887 negatively regulated cell proliferation and invasion in cervical cancer.

A large amount of evidence indicated that the combination of lncRNAs and miRNAs regulates the expression of target genes. Bioinformatics, luciferase reporter gene and pull-down assays predicted and confirmed that there was a binding site for miR-454-3p on linc00887. Subsequent functional experiments confirmed that linc00887 reversely regulates miR-454-3p level, and the re-expression of miR-454-3p can reverse the inhibition of linc00887 on cervical cancer. Moreover, we found that miR-454-3p was upregulated in Hela or C33A cells. In addition, miR-454-3p inhibitor significantly reduced the downregulation of linc00887 and promoted cell proliferation and invasion.

MiRNAs directly bind to the 3′-untranslated region (3′-UTR) of the target protein, which may cause further degradation of the gene or inhibit protein translation. In this study, FRMD6 was identified a target of miR-454-3p. FRMD6 is an Ezrin/Radixin/Moesin (ERM) family protein and is a human homologue of Drosophila extended (ex). Ex plays a role in parallel with Drosophila merlin upstream of the Hippo signaling pathway, which controls the proliferation, apoptosis, tissue regeneration and tumorigenesis of Drosophila [26]. Some studies have shown that FRMD6 inhibited the proliferation of human glioma cells, which is consistent with our tend in cervical cancer [27]. Bioinformatics and double-luciferase reporter gene assay predicted and confirmed that FRMD6 bound to miR-454-3p. We found that FRMD6 mRNA level was downregulated in Hela or C33A cells. Moreover, miR-454-3p inhibitor reversed the downregulation of FRMD6 protein level induced by linc00887 knockdown. FRMD6 was reported to be a key upstream component of the Hippo signaling pathway [28, 29], and Hippo signaling pathway is considered a key carcinogenic pathway for multiple tumors [28, 30, 31] or asthma treatment [32]. This Hippo pathway is one of the main conservative mechanisms controlling cell contact inhibition, organ size control and cancer development [33], and the downstream of the signaling pathway is transcriptional coactivator yorkie (Yki)/the transcriptional coactivator Yes-associated protein (YAP)/TAZ transcriptional activator [34]. The study of Hippo Yap pathway in head and neck squamous cell carcinoma showed that overexpression of PIK3CA (phosphatidylinositol 3-kinase catalytic subunit α) or the loss of FAT1 (fatty atypical cadherin 1) function could lead to the activation of yap [35]. Our research found that overexpression of FRMD6 enhanced the phosphorylation of MST1/2, LATS1/2 and TAZ proteins, and FRMD6 knockdown had the opposite function. In addition, subsequent functional assay certificated that miR-454-3p reversed the facilitation of phosphorylation of MST1/2, LATS1/2 and TAZ proteins in cervical cancer induced by linc00887 overexpression, which indicating that linc00887 inhibited the progression of cervical cancer via regulating FRMD6 expression.

## Conclusion

In conclusion, these findings indicated that Linc00887 sponging miR-454-3p inhibited the progression of cervical cancer through activating FRMD6-Hippo axis signaling pathway.

## Supplementary Information

**Additional file 1: Figure S1.** Linc00887 positively regulated expression of TIMP-1 and TIMP-2 and negatively regulated expression of MMP-2 and MMP-9. Vector (pcDNA3.1 empty vector), linc00887 (pcDNA-linc00887), scramble and linc00887 siRNA vectors were transfected into Hela cells, and then Western blot assay was used to detect the protein levels of TIMP-1, TIMP-2, MMP-2 and MMP-9 (A and B). **p < 0.01 versus Vector or Scramble.

## Data Availability

The datasets used and/or analyzed during the present study are available from the corresponding author on reasonable request.

## Abbreviations

lincRNA: Long intergenic noncoding RNA
HPV: Human papillomavirus
CIN: Cervical intraepithelial neoplasia
GAS5: Growth arrest special 5
miRNAs: MicroRNAs
FRMD6: FERM domain containing protein 6
TIMP-1/2: Tissue Inhibitor Of Matrix Metalloprotease-1/2
MMP-9/2: Matrix metalloproteinase-9/2
