# Supplementary material for: Linc00887 suppresses tumorigenesis of cervical cancer through regulating the miR-454-3p/FRMD6-Hippo axis
Source: Cancer Cell Int. 2021 Jan 7;21:33. doi: 10.1186/s12935-020-01730-w (PMC7792119; doi:10.1186/s12935-020-01730-w)
Supplement: Supplementary file 1 — Additional file 1: Figure S1. Linc00887 positively regulated expression of TIMP-1 and TIMP-2 and negatively regulated expression of MMP-2 and MMP-9. Vector (pcDNA3.1 empty vector), linc00887 (pcDNA-linc00887), scramble and linc00887 siRNA vectors were transfected into Hela cells, and then Western blot assay was used to detect the protein levels of TIMP-1, TIMP-2, MMP-2 and MMP-9 (A and B). **p < 0.01 versus Vector or Scramble. [file 12935_2020_1730_MOESM1_ESM.doc]

**
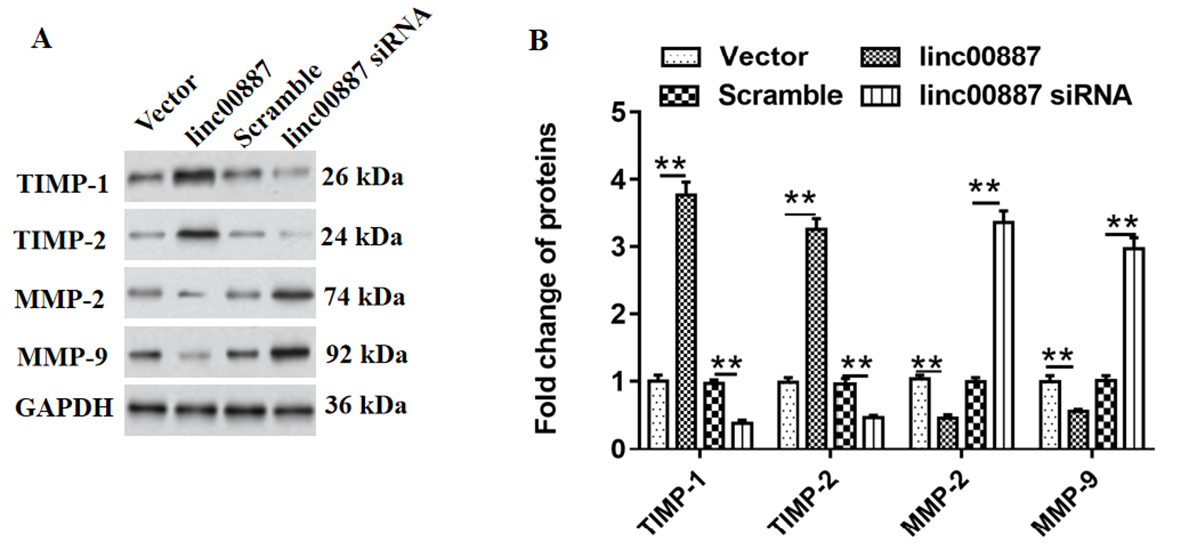
**

**Figure S1.** **Linc00887 positively regulated expression of TIMP-1 and TIMP-2 and negatively regulated expression of MMP-2 and MMP-9.** Vector (pcDNA3.1 empty vector), linc00887 (pcDNA-linc00887), scramble and linc00887 siRNA vectors were transfected into Hela cells, and then Western blot assay was used to detect the protein levels of TIMP-1, TIMP-2, MMP-2 and MMP-9 (A and B). ***p* < 0.01 versus Vector or Scramble.
